# Thermal Behaviour of Metakaolin/Fly Ash Geopolymers with Chamotte Aggregate

**DOI:** 10.3390/ma9070535

**Published:** 2016-06-30

**Authors:** Pavel Rovnaník, Kristýna Šafránková

**Affiliations:** Faculty of Civil Engineering, Brno University of Technology, Veveří 95, Brno 602 00, Czech Republic; kristynasaf@email.cz

**Keywords:** geopolymer, fly ash, metakaolin, high temperatures, chamotte, microstructure

## Abstract

Geopolymers are generally appreciated for their good resistance against high temperatures. This paper compares the influence of thermal treatment with temperatures ranging from 200 to 1200 °C on the mechanical properties and microstructure of geopolymers based on two different aluminosilicate precursors, metakaolin and fly ash. Moreover, the paper is also aimed at characterizing the effect of chamotte aggregate on the performance of geopolymers subjected to high temperatures. Thermal treatment leads to a deterioration in the strength of metakaolin geopolymer, whereas fly ash geopolymer gains strength upon heating. The formation of albite above 900 °C is responsible for the fusion of geopolymer matrix during exposure to 1200 °C, which leads to the deformation of the geopolymer samples. Chamotte aggregate improves the performance of geopolymer material by increasing the thermal stability of geopolymers via sintering of the aggregate particles with the geopolymer matrix in the contact zone.

## 1. Introduction

Geopolymers are inorganic materials with several superior properties which have been intensively studied for the last two decades as alternatives to Portland cement-based materials. The name ‘geopolymer’ generally refers to aluminosilicate materials synthesized at room temperature or slightly above via the alkali activation of reactive aluminosilicate precursors. The most commonly used aluminosilicates are metakaolin and fly ash from the high-temperature combustion of coal [1,2,3]; however, in recent years attention is also paid to other waste materials, such as rice husk ash [4], palm oil fuel ash [5], municipal solid waste incinerator fly ash [6], or red mud [7], and various natural pozzolans [8,9,10]. The structure of geopolymer can be either amorphous or crystalline, depending on the temperature of condensation. Amorphous geopolymers are produced at temperatures of up to 90 °C, while at higher temperatures the geopolymer is prone to form zeolite-like crystalline phases [11,12,13,14].

Geopolymers attract commercial attention not only for their superior mechanical properties and corrosion resistance, but also as fire-resistant materials. Geopolymer composites can be used as alternatives to organic polymer composites because they do not ignite, burn, or release any smoke during exposure to fire [15]. The binder remains amorphous over a wide range of temperatures; however, at temperatures over 1000 °C several crystalline species have been observed according to the chemical composition of the original geopolymer. The presence of kalsilite (KAlSiO_4_) and leucite (KAlSi_2_O_6_) have been proven in heat-treated samples of potassium geopolymers [16], while nepheline (NaAlSiO_4_), jadeite (NaAlSi_2_O_6_), and albite (NaAlSi_3_O_8_) have been observed in Na-based geopolymers [17,18,19]. These minerals are, thus, controlling factors for the fusion of geopolymer matrix. According to Davidovits [20], fusion temperature depends on the chemical composition of geopolymers and ranges from 950 to 1350 °C.

Thermal treatment of geopolymers is also followed by considerable shrinkage. Duxson et al. [21,22] showed that the overall thermal shrinkage is influenced greatly by Si/Al ratio and the type of alkali metal. The extent of thermal shrinkage decreases in the order Na > NaK > K for mixtures with Si/Al < 1.65, but the type of alkali ion has a negligible effect at higher Si/Al ratios. According to the composition, the linear shrinkage during thermal treatment up to 1000 °C can vary between 2% and 20% for geopolymer pastes, which is a very high value with respect to engineering applications. A possible way of mitigating thermal shrinkage is through the utilization of thermally-stable aggregate. Quartz and granite have been proven to reduce shrinkage to 1% but the disruptive phase change of quartz at 573 °C limits the working temperature range of such geopolymer composites [23,24,25]. It is, therefore, worth using artificial aggregates such as chamotte, crushed porcelain, or cordierite, which are fabricated at a very high temperature and have a low thermal expansion coefficient.

The motivation for the present work is to compare the thermal behaviour of geopolymers based on two different types of aluminosilicate, metakaolin and fly ash, and to investigate the influence of thermally-stable aggregate on the mechanical properties, shrinkage, and microstructure of both geopolymer composite materials. The positive effect of various artificially prepared aggregates on the engineering properties of thermally-treated alkali-activated slag composite was already presented in the author’s previous works [25,26,27]. Chamotte aggregate proved to have a very good stability in the alkaline environment of the slag matrix at very high temperatures. However, the chemical structure of geopolymers is completely different from the one observed in alkali-activated slag, and the content of alkalis, which is crucial for the thermal stability, is much higher [28]. One of the objectives of this work was to investigate the performance of thermally-stable aggregates in such highly-alkaline matrix and, based on the previous experience, chamotte was chosen as the most suitable option for this study.

## 2. Experimental Part

### 2.1. Materials

**Figure 1:**
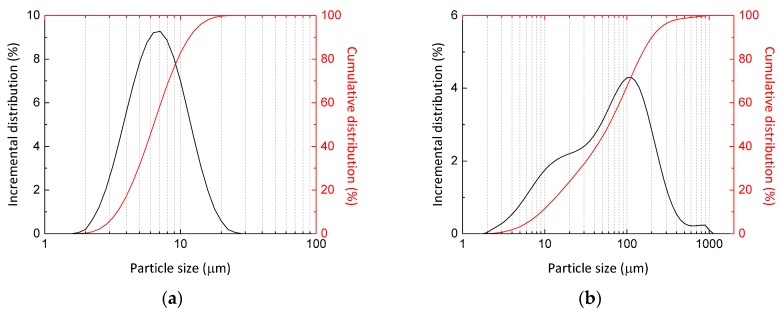
Particle size distribution of metakaolin Mefisto K05 (**a**) and low-calcium fly ash (**b**).

The geopolymers used in the tests were based either on metakaolin or fly ash. Metakaolin (MK) was supplied by ČLUZ (Nové Strašecí, Czech Republic) under the brand name Mefisto K05. Fly ash (FA) was obtained from disposal waste resulting from the combustion of pulverised coal in a coal-fired furnace and is classified as Class F fly ash according to the ASTM C618 standard. The particle size distribution of both aluminosilicates obtained by laser granulometry is given in Figure 1. According to XRD analysis both aluminosilicates contained traces of quartz and mullite as the only crystalline phases present. A sodium silicate solution with either SiO_2_/Na_2_O = 1.4 (WG 1) or SiO_2_/Na_2_O = 2.0 (WG 2) was used as an activator. Chamotte (grain size 0–5 mm) was used as a heat resistant aggregate for the preparation of the geopolymer mortars. The main mineralogical phases found in chamotte aggregate using XRD analysis are quartz, cristobalite, and mullite. The chemical compositions of the materials used are presented in Table 1. The composition of solids was determined by means of X-ray fluorescence analysis and the composition of water glass solutions was obtained by the classical titration method [29].

### 2.2. Sample Preparation

Pastes were prepared by mixing the aluminosilicate precursor with an activating solution and additional water in a planetary mixer for 5 min in order to prepare a homogenous mixture. The metakaolin was activated by the WG 1 solution, whereas the fly ash was activated by WG 2. In case of mortars, the chamotte aggregate was added to the geopolymer paste and mixed for further 3 min. The amount of additional water was adjusted to obtain a similar consistency of both geopolymer types. The mix compositions of the tested geopolymer pastes and mortars are presented in Table 2. The amount of chamotte aggregate was selected based on our previous investigations concerning similar materials [24,25,26,27]. The mixtures were cast into 40 × 40 × 160 mm prismatic moulds and sealed with PE film to prevent the mixing water from evaporating, and stored under laboratory conditions (22 ± 2 °C; relative humidity ϕ = 45% ± 5%). After seven days, the hardened specimens were demoulded and stored in plastic bags under laboratory conditions for another 21 days. The prepared geopolymer specimens were then heated in a Muffle furnace to temperatures of 200, 400, 600, 800, 1000, and 1200 °C at a constant heating rate of 5 °C min^−1^. The specimens were kept at the given temperature for 1 h and then allowed to cool down to room temperature.

### 2.3. Testing Procedure

Bulk density of the specimens was measured following the procedure described for cement mortars in the EN 196-1 standard [30]. Linear shrinkage of heat treated specimens was determined as a percentage change in the longest dimension of the specimen. Specimens were also tested for their residual mechanical properties, which were compared with those obtained for unheated samples at the age of 28 days. Mechanical tests were carried out on Tonindustrie Prüftechnik D-1000 testing machine (Tonitechnik, Berlin, Germany) with the measuring range 0–200 kN. Flexural strengths were determined using a standard three-point-bending test and compressive strengths were measured on the far edge of each of the two residual pieces obtained from the flexural test according to the EN 196-1 standard. For each temperature a set of three specimens was used.

Pore distribution was evaluated by means of mercury intrusion porosimetry (MIP) analysis, which was conducted on the paste samples using a Micromeritics Poresizer 9310 porosimeter that can generate a maximum pressure of 207 MPa and can evaluate a theoretical pore diameter of 0.006 μm. The MIP test is performed in two steps. In the first (low pressure) step, gases are evacuated, the sample holder is filled with mercury and porosimetry is performed from about 7 to 179 kPa. In the second (high-pressure) step, pressures between 414 kPa and 207 MPa are reached. The contact angle and surface tension assumed for all tests were 130° and 485 mN·m^−1^, respectively.

X-ray diffraction analyses were carried out using a Bruker D8 Advance system equipped with a Cu tube (λ_Kα_ = 1.54184 Å). The instrument is also equipped with an Anton Paar HTK 16 temperature attachment which facilitates measurements at up to 1600 °C. Θ–Θ reflection geometry was used. The evaluation of X-ray scans was performed using Diffrac software. Micrographs of the alkali- activated slag pastes were taken on JEOL JSM-840 (JEOL, Tokyo, Japan) and partly on a Tescan MIRA3 XMU (Tescan, Brno, Czech Republic) scanning electron microscope. The experiments were carried out on dry samples that were sputtered with carbon.

## 3. Results

**Figure 2:**
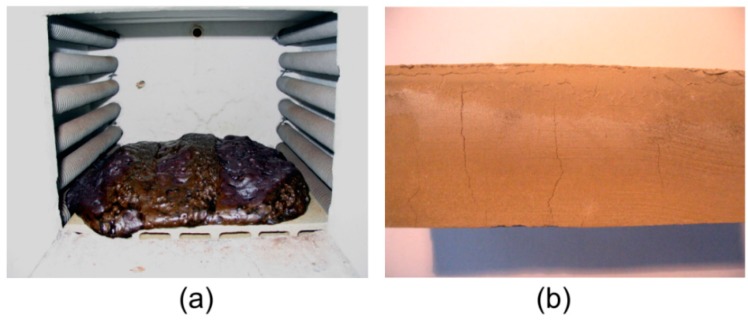
Appearance of FA geopolymer after thermal treatment: (**a**) flushing of FA paste specimens after heating to 1000 °C; and (**b**) transverse cracks on FA paste specimens after heating to 600 °C.

Both the metakaolin and the fly ash geopolymers underwent structural changes when they were exposed to very high temperatures. The main problem occurred when the geopolymer pastes were exposed to 1200 °C. Such a temperature is high enough for the fusion of the geopolymer matrix, so the fly ash geopolymer was completely damaged and the metakaolin geopolymer specimens were distorted. As a result, no mechanical tests could have been performed for pastes exposed to 1200 °C (Figure 2a). In the case of the mortars with chamotte aggregate, only metakaolin geopolymer was able to withstand the temperature of 1200 °C without suffering significant damage.

### 3.1. Mechanical Properties

**Figure 3:**
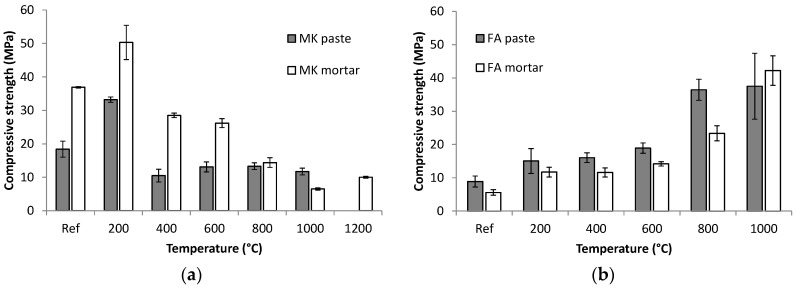
Residual compressive strengths of metakaolin (**a**) and fly ash (**b**) geopolymer pastes and mortars after exposure to high temperatures. Standard deviations are depicted as error bars.

The properties of the investigated geopolymer pastes and mortars after high-temperature treatment were compared with a reference material that was treated at ambient temperature. Their compressive strength versus temperature is presented in Figure 3.

In the case of MK geopolymer, the mortar with chamotte aggregate exhibited much higher strength than the corresponding paste. Exposure to 200 °C caused the material to be strengthened by 80% for the paste and 36% for the mortar. Such an effect is quite common even for cement-based materials and can be attributed to the stiffening of the gel and the increase in surface forces between the gel particles due to the release of adsorbed moisture [31,32] or to the promotion of polycondensation between chain-like geopolymer gels [33]. At higher temperatures, compressive strengths decreased again, which was more pronounced in the case of MK geopolymer paste, where only a small variation in strength between 400 and 1000 °C was observed. However, residual strengths were still 12 MPa (64%) for MK paste at 1000 °C, and 10 MPa (27%) for MK mortar at 1200 °C.

FA geopolymer surprisingly showed a different trend in compressive strength values when exposed to elevated temperatures. The unheated reference mixtures had relatively low strength, due to slower reaction kinetics of the fly ash, but this gradually increased with temperature. Residual strengths after heating to 1000 °C were 37 MPa (422%) and 42 MPa (757%) for FA paste and FA mortar, respectively. In contrast to MK geopolymer, FA paste exhibited higher compressive strength values than the corresponding mortar with chamotte aggregate.

**Figure 4:**
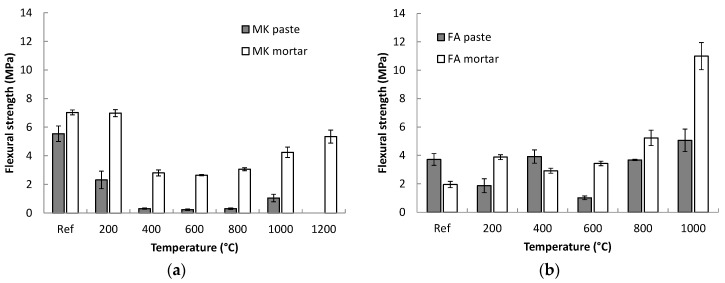
Residual flexural strengths of metakaolin (**a**) and fly ash (**b**) geopolymer pastes and mortars after exposure to high temperatures. Standard deviations are depicted as error bars.

The residual flexural strengths of the tested geopolymers after high temperature loading are presented in Figure 4. The changes in flexural strengths after exposure to increasing temperatures partially followed the trends observed for the corresponding compressive strengths. In the case of MK geopolymer paste, the strength rapidly dropped to only 0.3 MPa during heating to 400 °C. Such a decrease corresponded to the level of internal cracking of the geopolymer paste during heating. Similar behaviour was also observed for MK mortar, but the drop in strength was not so critical because the aggregate is able to mitigate the tensile stress caused by shrinkage of the geopolymer binder. Above 1000 °C, the strength increased again but did not regain the reference value.

Fly ash geopolymer showed no obvious trend in flexural strength values after exposure to high temperatures. There was a decrease in strength for FA paste at 200 and 600 °C but generally flexural strength remained around 4 MPa. The considerable drop in strength at 600 °C is associated mainly with the formation of large and wide cracks across almost the whole cross section of the specimen (Figure 2b). However, a substantial increase in strength was observed for FA mortar after exposure to temperatures of 800 °C and higher. The flexural strength of the FA mortar heated to 1000 °C was 5.6 times higher than the reference value.

### 3.2. Bulk Density and Shrinkage

**Figure 5:**
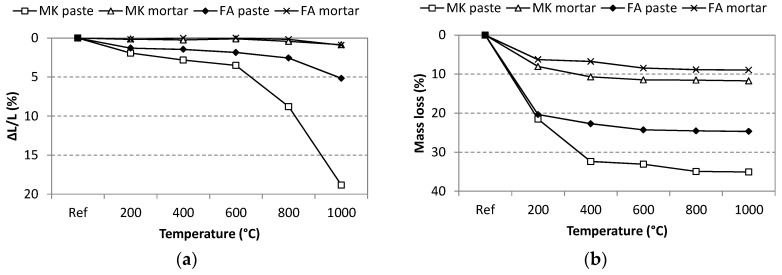
Thermal shrinkage (**a**) and mass loss (**b**) of geopolymer samples.

It was observed that exposure to elevated temperatures led samples to undergo considerable volume changes, which were expressed via the linear shrinkage of the geopolymer specimens (Figure 5). The shrinkage of both geopolymer pastes was more significant than in the case of the corresponding mortars. The shrinkage of the mortars was very low and did not exceed 1% at any temperature. The most considerable shrinkage was achieved for the MK paste. The key temperature interval at which the enormous shrinkage occurred was between 600 and 800 °C, and the maximum shrinkage of 18% was observed at 1000 °C. This volume contraction is connected with microstructural changes in the geopolymer matrix. The FA paste also exhibited increased shrinkage, but a maximum of 5%, which is comparable to the shrinkage of alkali-activated slag or Portland cement paste [34].

**Figure 6:**
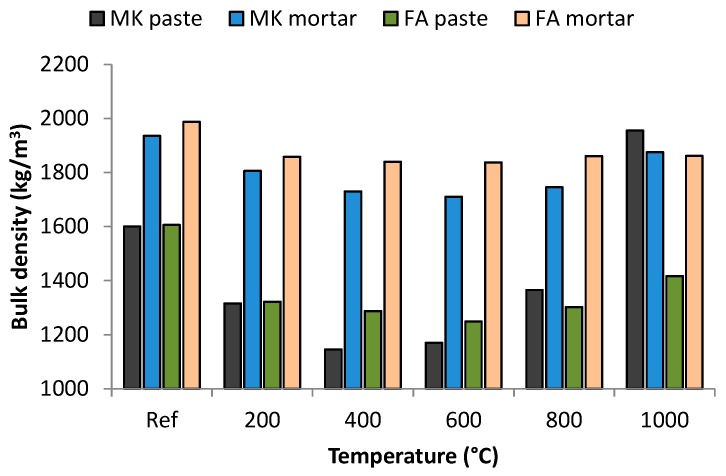
Bulk density of geopolymer samples exposed to elevated temperatures.

Despite the severe shrinkage of the specimens, no essential change in bulk density was observed, except in the case of MK paste (Figure 6). This can be explained by a simultaneous shrinkage and mass loss of heated geopolymers (Figure 5). A decrease in the Ref–400 °C range can be recognised and is attributable to the loss of physically- and chemically-bound water [26]. Since no mass loss was observed at temperatures higher than 800 °C, the shrinkage predominates over the mass loss and, therefore, in the case of MK paste heated to 1000 °C the bulk density even exceeded the value of the reference sample. The chamotte used as aggregate is a heat resistant material with a low thermal expansion coefficient (5.2 × 10^−3^) [35], so the geopolymer mortars that contain it are not so prone to changes in volume.

### 3.3. SEM Analysis

**Figure 7:**
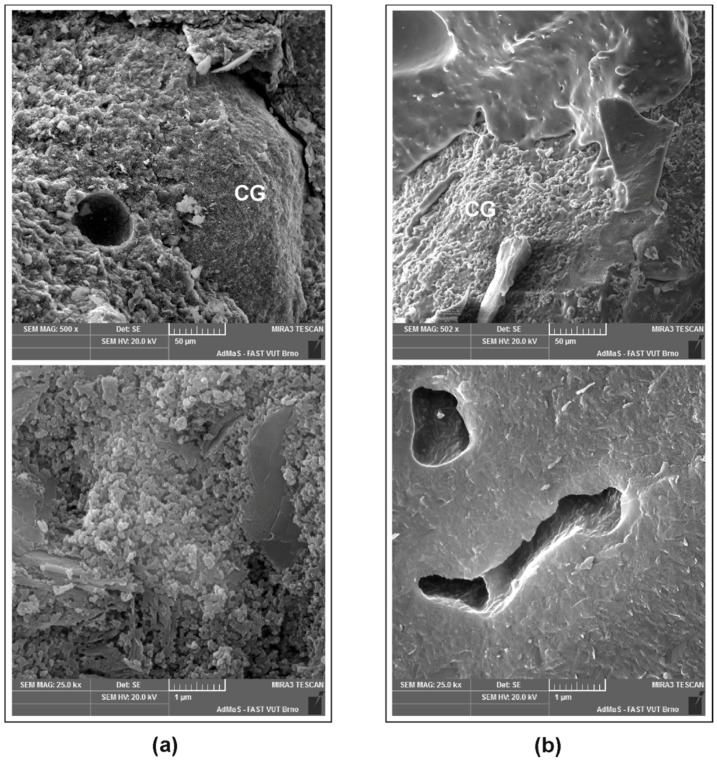
Micrographs of MK mortar treated: (**a**) at room temperature; and (**b**) after heating to 1000 °C (CG = chamotte grain).

The main structural changes were observed by means of scanning electron microscopy. Figure 7 shows the differences in the morphologies of the reference and heated MK mortars as expressed on the fracture surfaces of the specimens. The microstructure of the MK matrix is very porous and displays residues of lamellar metakaolin particles; the bond to the fireclay aggregate is quite loose. After exposure to 1000 °C, the geopolymer matrix sintered to form a very compact substance in which only larger pores remained. However, this process did not lead to the strengthening of the bond between the matrix and aggregate.

**Figure 8:**
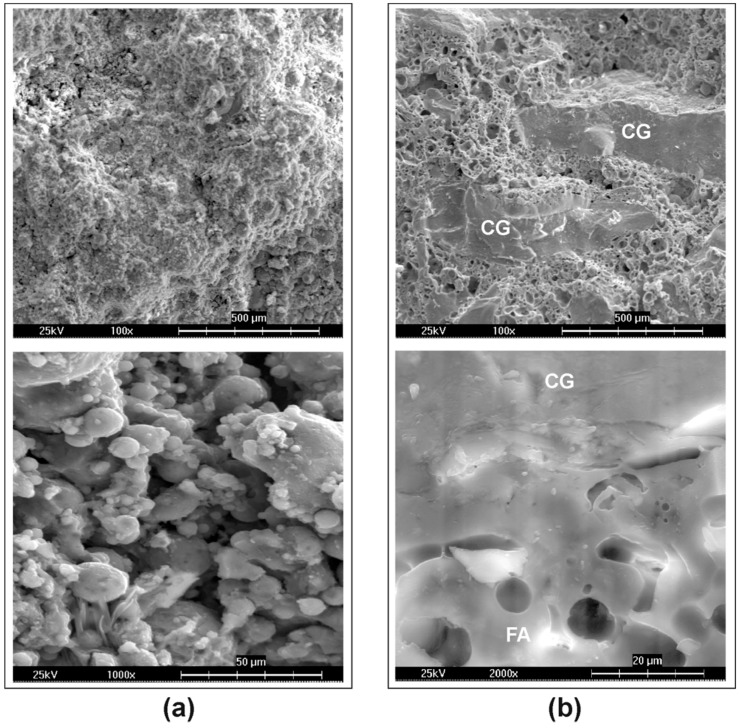
Micrographs of the FA mortar treated: (**a**) at room temperature; and (**b**) after heating to 1000 °C (CG = chamotte grain).

Micrographs of the reference FA geopolymer (Figure 8a) show that the fly ash grains are very loosely connected to each other, leaving quite a lot of space between them that can be associated with large capillary pores. This structure contributes to the rather low strength of FA geopolymer. Heating to 1000 °C caused the fusion and sintering of fly ash grains and also produced a good bond between the geopolymer matrix and the chamotte aggregate (Figure 8b). As a result, fracture of the fly ash, as well as chamotte grains, was observed, and the fracture surface is smoother.

### 3.4. Pore Structure

**Figure 9:**
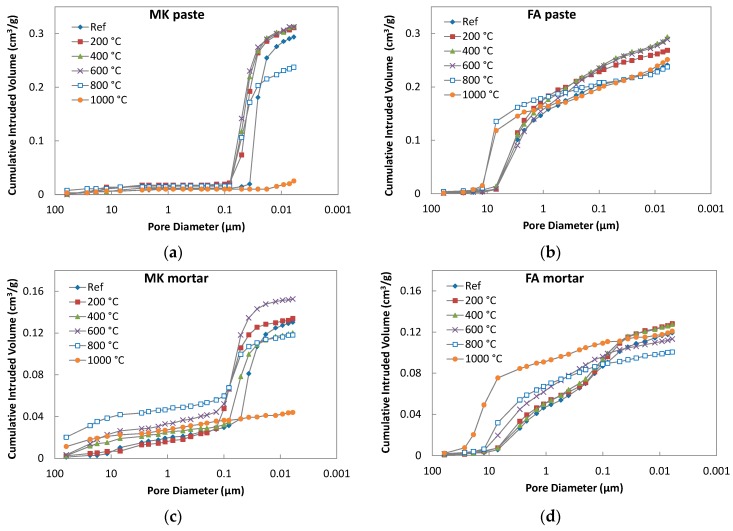
Cumulative intruded volume of geopolymer pastes and mortars subjected to temperatures of 200–1000 °C. (**a**) MK paste; (**b**) FA paste; (**c**) MK mortar; (**d**) FA mortar.

The pore size distributions of geopolymer pastes and mortars subjected to high temperatures observed by means of mercury intrusion porosimetry are presented in Figure 9. There is a substantial difference in the pore system between the FA and MK geopolymers. In the FA based material, a large volume of pores is associated with capillary pores of between 1 and 10 µm in size while, in the case of the MK geopolymer, the majority of pores are smaller than 100 nm. The use of non-porous chamotte aggregate caused a general decrease in the total intruded volume of pores in mortars, but they had an opposite effect on the pore distribution. In the case of FA mortar, smaller pores tend to predominate, whereas the addition of aggregates increased the number of pores larger than 100 nm in geopolymer mortar prepared from metakaolin. The effect of high-temperature treatment on the porosity of geopolymers was more pronounced for the metakaolin type. During the heating of FA geopolymer, the volume of pores between 0.1 and 10 µm increased to the detriment of smaller pores; however, it had almost negligible effect on the total porosity. In the case of MK geopolymer, the total intruded volume slightly increased to 600 °C, but then rapidly dropped. Only very large pores remained in the geopolymer matrix at 1000 °C, implying that a kind of sintering process occurred.

### 3.5. High-Temperature X-ray Diffraction Analysis (HT-XRD)

**Figure 10:**
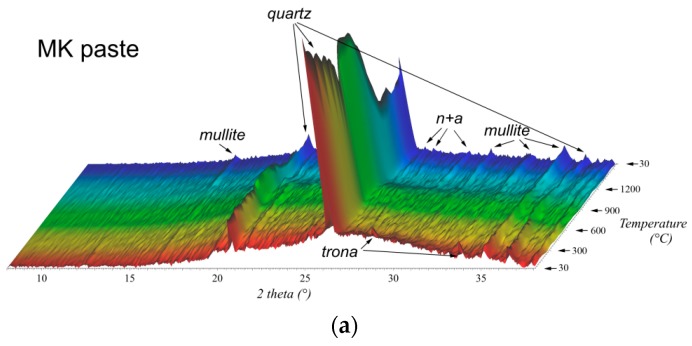

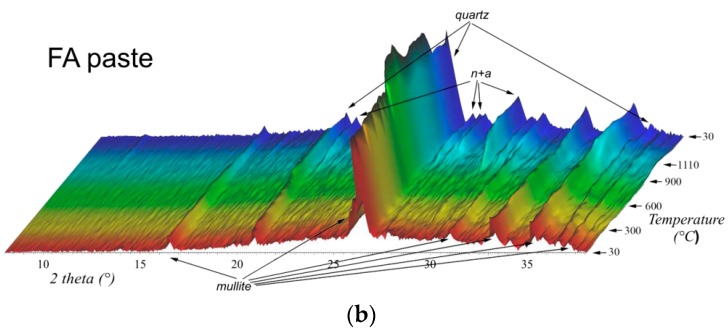
High-temperature XRD patterns for MK paste (**a**) and FA paste (**b**) measured in the temperature range of 30–1200 °C (n + a = nepheline + albite).

High-temperature XRD analysis was employed to observe the mineral transitions in the geopolymer pastes during exposure to high temperatures (Figure 10). A broad hump centred at 27° of 2θ in the XRD pattern is associated with amorphous geopolymer gel. The main features in the XRD pattern belong to quartz and mullite, which are present as minor components in both aluminosilicate precursors. MK geopolymer also contained traces of trona resulting from the carbonation of free alkalis that did not take part in the geopolymerisation reaction. During heating only one new crystalline phase, which was attributed to nepheline and albite, appeared in the XRD patterns. Its signals started rising at 900 °C and were slightly more pronounced in the case of FA paste. MK geopolymer contained predominantly nepheline, whereas in FA geopolymer albite was the dominating phase.

## 4. Discussion

The performance of the two investigated types of geopolymer material differed greatly, as did the effect of chamotte aggregate on their properties. Generally, FA geopolymer exhibited worse mechanical properties than MK geopolymer because of the weaker contacts between the FA grains and the apparently lower reaction degree that can be attributed on one hand to slower reaction kinetics of fly ash and, on the other hand, to its larger particles and, thus, lower specific surface area. The lower compressive strength of the FA geopolymer can also be associated with its higher pore volume in the range of large capillary pores, whereas a major part of MK geopolymer porosity involves pores smaller than 100 nm. Chamotte was able to improve the compressive strength by 100% and flexural strength by 27% in the case of MK mortar due to the good bond between the MK matrix and the aggregate grains. However, no improvement was observed for FA mortar, showing that the bond between fly ash and chamotte is very weak.

Very high temperatures caused considerable changes in the microstructure and properties of both geopolymer materials. While MK geopolymer showed a slight improvement in mechanical properties after exposure to 200 °C and rapid deterioration at higher temperatures, FA geopolymer gained strength when heated. Heating of MK paste was followed by enormous shrinkage of the geopolymer matrix, which resulted in microcracking and a complete loss of flexural strength. The addition of chamotte aggregate helped to mitigate the shrinkage of MK paste and, thus, the drop in strength as well. The effect of chamotte on the mechanical properties of FA geopolymer was not so fundamental, although it reduced shrinkage to the same extent as in the case of MK mortar. The reason for the improvement in strength lies in the successive hydrothermal reaction of the FA particles and their subsequent fusion due to the high alkali content, which resulted in the formation of a ceramic bond between the FA particles, as well as the geopolymer matrix and chamotte aggregates. 

The high content of Na^+^ ions in geopolymer matrices leads to the crystallization of nepheline and albite at temperatures over 900 °C, which are responsible for the strengthening of both geopolymers. These observations are in accordance with previous data [17,18]. Since the melting point of albite is in the range of 1100–1120 °C [36], heating to 1200 °C caused severe deformation of the specimens, and in the case of FA paste it even brought about the flushing of the material due to the higher content of albite in FA geopolymer, although the total alkali content was lower than in MK paste.

In geopolymer mortars, alkalis also attack the surface layer of chamotte aggregate, which leads to the lowering of its fusion temperature. As a result, the contact zone between aggregate grain and matrix sinters, contributing to the increased compactness of the structure. Such a process plays an important role in the reduction of shrinkage and the stabilization of geopolymer mortars at very high temperatures. This was proven especially in the case of MK mortar, which resisted exposure to 1200 °C without significant damage, meaning mechanical tests could have been performed. In contrast to other aggregates, such as quarried quartz sand, chamotte is produced at approx. 1350 °C and has a very low thermal expansion coefficient [25,35]; it is, therefore, one of the best aggregates that can be used in building materials subjected to high temperatures as it does not influence their performance, and sometimes even improves it.

## 5. Conclusions

This paper presents an investigation of the behaviour of geopolymer materials based on the combination of metakaolin and fly ash with chamotte aggregate. The materials were subjected to temperatures ranging from 200 to 1200 °C. The following conclusions have been drawn from the experimental results and previous discussion:

- Geopolymer based on metakaolin showed better mechanical properties compared to FA geopolymer when they are treated under laboratory conditions. This difference can be attributed to slower reaction kinetics of fly ash, as well as its larger particles and, therefore, curing time of 28 days might not have been sufficient to provide complete geopolymerisation. However, exposure to high temperatures has a different effect on each of the geopolymers. While MK geopolymer suffers from a substantial deterioration in its mechanical properties, FA geopolymer gains strength upon heating.
- Chamotte aggregate improves the performance of MK geopolymer because it considerably reduces shrinkage of the geopolymer binder during heating. Unfortunately, it has a slightly negative effect on the compressive strength of FA geopolymer. However, at temperatures over 800 °C chamotte is attacked by alkalis, which causes partial sintering in the contact zone between the aggregate and the geopolymer matrix. This effect plays a substantial role in the improvement of residual mechanical properties after exposure to 1000 °C and helps MK mortar to resist higher temperatures without damage.
- Free alkalis present in geopolymer matrix lead to the formation of nepheline and albite at temperatures above 900 °C. Due to the relatively low melting point of albite, its occurrence is responsible for the fusion of geopolymer at 1200 °C and the deformation of the structure. Despite the lower content of alkalis in the matrix it is more pronounced for FA-based material because of enhanced albite formation.

## Figures and Tables

**Table 1:** Chemical compositions of the materials used in the mixtures.

Composition (wt. %)	Metakaolin	Fly Ash	Chamotte	WG 1	WG 2
SiO_2_	55.01	49.82	53.95	24.90	23.22
Al_2_O_3_	40.94	24.67	42.15		
Fe_2_O_3_	0.55	7.05	1.25		
CaO	0.55	3.91	0.13		
MgO	0.14	2.68	0.18		
S_total_	0.34	0.91			
Na_2_O	0.09	0.70	0.05	18.47	12.25
K_2_O	0.60	2.78	0.75		

**Table 2:** Mix proportions of metakaolin/fly ash geopolymer paste and mortar and Si/Al and Na/Al ratios in the corresponding geopolymer mixture.

Components	MK Paste	MK Mortar	FA Paste	FA Mortar
Metakaolin (g)	1000	1000		
Fly ash (g)			1000	1000
WG 1 (g)	849	849		
WG 2 (g)			437	437
Chamotte (g)		3000		3000
Water (g)	267	343	132	237
Si/Al	1.50		2.06	
Na/Al	0.64		0.53	
